# In-vivo effects of intraocular and intracranial pressures on the lamina cribrosa microstructure

**DOI:** 10.1371/journal.pone.0188302

**Published:** 2017-11-21

**Authors:** Bo Wang, Huong Tran, Matthew A. Smith, Tigran Kostanyan, Samantha E. Schmitt, Richard A. Bilonick, Ning-Jiun Jan, Larry Kagemann, Elizabeth C. Tyler-Kabara, Hiroshi Ishikawa, Joel S. Schuman, Ian A. Sigal, Gadi Wollstein

**Affiliations:** 1 Department of Ophthalmology, University of Pittsburgh School of Medicine, University of Pittsburgh Medical Center Eye Center, Eye and Ear Institute, Ophthalmology and Visual Science Research Center, Pittsburgh, Pennsylvania, United States of America; 2 Department of Bioengineering, Swanson School of Engineering, University of Pittsburgh, Pittsburgh, Pennsylvania, United States of America; 3 New York University Langone Eye Center, New York University School of Medicine, New York, New York, United States of America; 4 Department of Neurosurgery, University of Pittsburgh School of Medicine, Pittsburgh, Pennsylvania, United States of America; Bascom Palmer Eye Institute, UNITED STATES

## Abstract

There is increasing clinical evidence that the eye is not only affected by intraocular pressure (IOP), but also by intracranial pressure (ICP). Both pressures meet at the optic nerve head of the eye, specifically the lamina cribrosa (LC). The LC is a collagenous meshwork through which all retinal ganglion cell axons pass on their way to the brain. Distortion of the LC causes a biological cascade leading to neuropathy and impaired vision in situations such as glaucoma and idiopathic intracranial hypertension. While the effect of IOP on the LC has been studied extensively, the coupled effects of IOP and ICP on the LC remain poorly understood. We investigated in-vivo the effects of IOP and ICP, controlled via cannulation of the eye and lateral ventricle in the brain, on the LC microstructure of anesthetized rhesus monkeys eyes using the Bioptigen spectral-domain optical coherence tomography (OCT) device (Research Triangle, NC). The animals were imaged with their head upright and the rest of their body lying prone on a surgical table. The LC was imaged at a variety of IOP/ICP combinations, and microstructural parameters, such as the thickness of the LC collagenous beams and diameter of the pores were analyzed. LC microstructure was confirmed by histology. We determined that LC microstructure deformed in response to both IOP and ICP changes, with significant interaction between the two. These findings emphasize the importance of considering both IOP and ICP when assessing optic nerve health.

## Introduction

The lamina cribrosa (LC), a fenestrated connective tissue meshwork located in the optic nerve head, plays an important role in blinding diseases.[1] The LC contains pores through which all retinal ganglion cell axons pass on their way to the brain. As such, the LC is sensitive to the mechanical pressures that surround it, intraocular and intracranial pressure. Mechanical deformation of the LC, studied in the context of elevated intraocular pressure (IOP) in glaucoma, has been shown to cause a biological cascade–including reduced axoplasmic transport of neurotrophic factors, tissue hypoxia, and glial cell activation[1–4]–that results in neuronal cell death. Furthermore, in states of elevated intracranial pressure (ICP), such as idiopathic intracranial hypertension, it has been shown that neurotrophic factors are blocked at the level of the LC.[5] Despite evidence of the common role of IOP and ICP on the LC, their effects have largely been studied separately, which does not account for the potential counter effect of the opposing pressures.[6–10]

IOP is the main risk factor in glaucoma, the second leading cause of irreversible blindness worldwide.[1,4] Recently, there is increasing evidence for the role of ICP in the disease.[6,11–14] A few studies reported significantly lower ICP in subjects with glaucoma compared with healthy subjects.[6,8] Furthermore, significantly higher ICP was recorded in subjects with ocular hypertension (no functional glaucomatous damage despite elevated IOP) compared with healthy eyes.[6,12] In an animal model, extended reduction in ICP resulted in ocular neural tissue loss in half of the monkeys.[8] These findings suggest that both IOP and ICP may play an important role in the disease process.

However, despite the LC’s role in glaucoma and diseases of altered ICP, limited information is available on in-vivo deformation of the LC as a response to acute IOP and ICP modulation. Previous study in a dog model demonstrated that the optic nerve head (ONH) surface deformations occurred at a range of IOP and ICP pressure differences.[15] While a previous work assessed the effects of IOP on the LC microstructure,[16] no information is available on the effects of both IOP and ICP on LC microstructure and the interactions between the pressures. The interaction between the pressures is crucial as many different IOP and ICP combinations can result in the same translaminar pressure difference. Studying the LC microstructure, such as the morphology of the LC beam and pores via parameters such as beam thickness and pore diameter, is important in order to determine the biomechanics of the tissue before remodeling changes occurring in response to the chronic conditions.[17,18] Only a thorough understanding of the acute effects of IOP and ICP modulations would enable determining the role of remodeling on the disease process. In-vivo experimental design has the key advantage of quantifying the pressure effects in a physiological state, with realistic conditions of tissue stiffness, blood pressures and other potentially contributing factors. These are difficult, perhaps impossible, to replicate in ex-vivo experiments or simulations.

The purpose of this study was to determine how both IOP and ICP affect the LC three-dimensional microstructure in a living primate eye. We hypothesize that both IOP and ICP will cause changes in the LC microstructure.

## Methods

### Animals

Five healthy, adult, rhesus macaque monkeys (Macaca mulatta) were used for this experiment. All procedures in this study were approved by the University of Pittsburgh’s Institutional Animal Care and Use Committee (IACUC) and adhered to both the guidelines set forth in the National Institute of Health’s Guide for the Care and Use of Laboratory Animals and the Association of Research in Vision and Ophthalmology (ARVO) statement for the use of animals in ophthalmic and vision research. This cohort included two females (Monkey 1—age 12 years, Monkey 2 –age 15) and three males (Monkey 3 –age 14 years, Monkey 4 –age 8.5, Monkey 5 –age 8). Both eyes of Monkey 5 were used to characterize the reproducibility of behavior between eyes in the same animal.

Animals were cared for and housed using the National Institutes of Health (NIH) Guide for the Care and Use of Laboratory Animals. A detailed protocol for care, including details on enrichment and surgical specifics, was approved by the Institutional Animal Care and Use Committee (IACUC) of the University of Pittsburgh. To highlight the environmental enrichment and housing, each monkey was housed either with or near other monkeys. Monkeys were given access to free water and given a variety of enrichments daily which included but not limited to visual, audio, taste, and textures as well as encouraged normal primate behavior of grooming and foraging.

### Experimental setting

**Fig 1:**
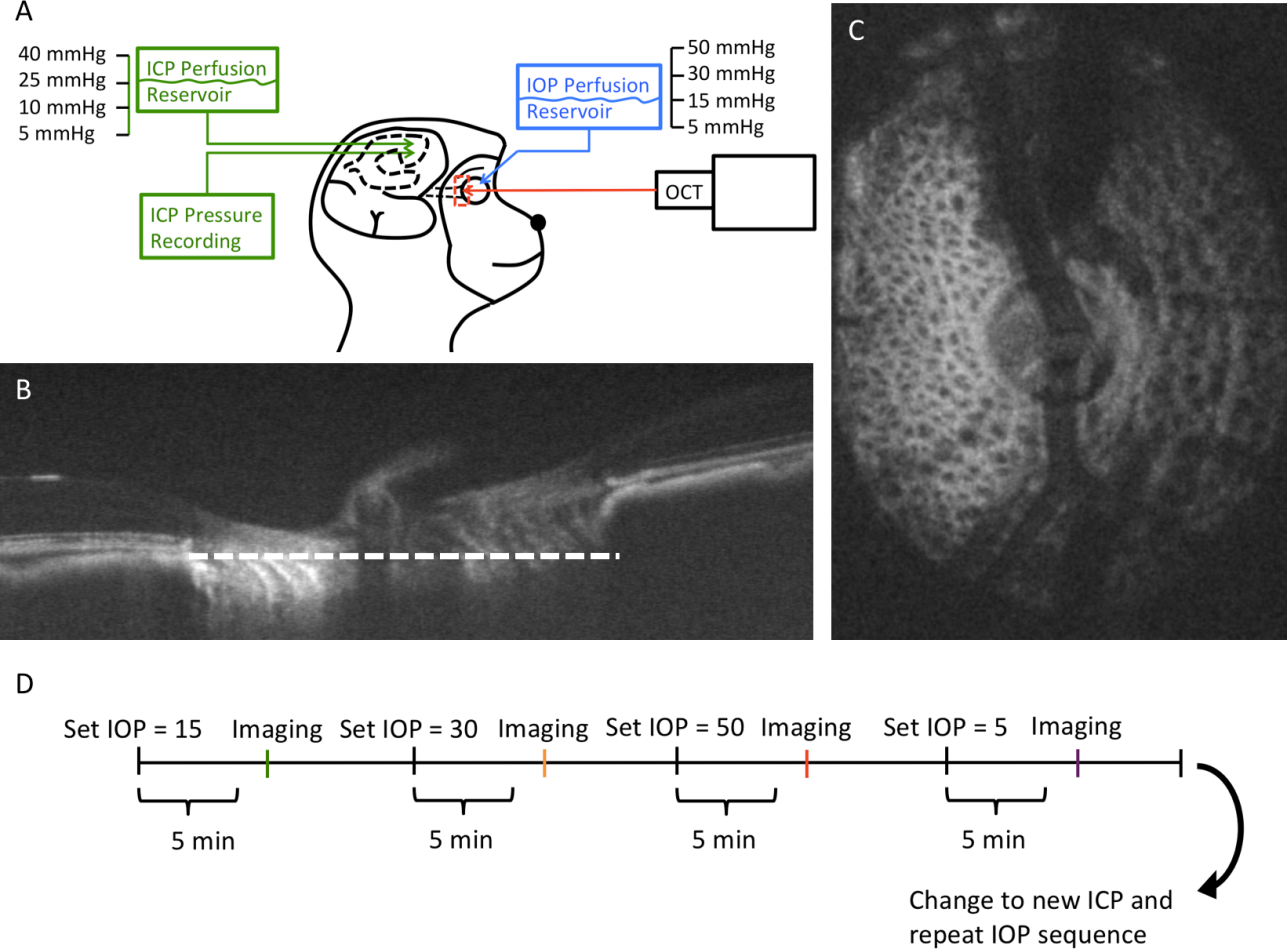
**(A) Diagram of the experimental setup**. Intraocular pressure (IOP) and intracranial pressure (ICP) were controlled using a gravity-based perfusion system. OCT imaging of the lamina cribrosa (LC) (red box) was performed after altering IOP and/or ICP. (B) A sagittal slice of the OCT volume. White dotted line denotes the plane of the (C) enface view of the ONH. (D) At every given ICP, IOP was altered and the ONH was imaged after allowing the tissue to stabilize for 5 minutes at every IOP condition. After completing all IOP conditions, a new ICP was set and the IOP conditions repeated.

The research design is summarized in Fig 1. The primates were scanned with their head upright and the rest of their body lying prone on a surgical table. The animals were anesthetized and OCT scans of the ONH region were acquired at baseline and at each pressure setting. ICP was adjusted and then IOP was modulated in the various pressure settings while acquiring OCT images at each setting. 5 minutes were given after changing pressure before OCT scanning was performed to reduce the viscoelastic effect on the eye[19]. After completing all IOP modulations, ICP was adjusted to a different pressure and the IOP modulation was repeated.

### Anesthesia

Animals were anesthetized with ketamine (20 mg/kg) and midazolam (0.25 mg/kg) and then intubated and maintained with isoflurane anesthesia (1–3%) for the duration of the experiment. Prior to imaging, animals were paralyzed using vecuronium bromide (2mg/hr) to reduce ocular movements during scanning, and were artificially ventilated to maintain an end-tidal CO_2_ of approximately 35mmHg. Euthanasia was performed using Somnasol (Henry Schein, Melville, NY) dosed at 85mg/kg.

### Pressure control

IOP was controlled via gravity-based perfusion through a 27-gauge needle inserted into the anterior chamber after thorough irrigation of the cannula to remove all air bubbles. A saline reservoir was raised above the height of the globe to set the IOP (5, 15, 30 and 50mmHg for Monkeys 1–3; 5, 15, 30, 40 for Monkeys 4–5). The lateral ventricle was cannulated with a lumbar catheter (Medtronic, Minneapolis, MN), also attached to a separate saline reservoir and thoroughly irrigated, to control ICP. The height of the reservoir was adjusted to achieve a target ICPs of 5, 10, 25 and 40mmHg. ICP was simultaneously and continuously measured with a fiber-optic pressure sensor inserted into the parenchyma of the brain (ICP EXPRESS monitoring system; DePuy Synthes, Raynham, MA). Before using the pressure transducer it was calibrated while submerged in saline solution. The precision of the IOP and ICP values were within the range of +/-1mmHg with fluid added or withdrawn to reach target pressure.

### OCT imaging

The pupils were dilated using tropicamide ophthalmic solution 0.5% (Bausch & Lomb, Rochester, NY) and the animals were scanned with their body in the prone position and head held upright and facing the OCT device. A rigid gas permeable contact lens (Boston EO, Boston, MA) was fitted to each scanned eye to improve image quality. The eyes were kept open using a wire speculum and the cornea was irrigated with artificial tears every 5 minutes. All eyes were scanned 4 times in each pressure setting in a 5mm x 5mm x 2mm volume (512 x 512 x 1024 samplings) centered on the ONH using spectral-domain OCT device (Bioptigen Spectral Domain Ophthalmic Imaging System, Research Triangle, NC) with a scan rate of 20,000 A-scans/second modified with a broadband superluminescent diode (Superlum, Dublin, Ireland; λ = 870 nm, Δλ = 200 nm) light source.

Images were acquired from six eyes of the five animals and scans with poor visibility of the LC (defined below) were removed from the analysis. Thirty-five out of 52 pressure conditions had visible LC microstructure, which were then used for microstructure analysis. The number of pores analyzed per eye ranged from 29 to 44 with a relatively small variability in each eye between the various pressure conditions.

### Image analysis

Images were subjectively inspected and those with poor LC microstructure visualization were discarded from analysis. The remaining images were subjectively graded for image quality. Quality was defined based on the ability to clearly differentiate beam and pore structure in the LC microstructure, as can be seen in S1 Fig. An experienced observer masked to the experimental setting in which the images were acquired determined the image quality on a scale from 1–3, with 1 being the worst and 3 being the best quality. A relatively high rate of poor image quality were detected at high ICP (S2 Fig).

**Fig 2:**
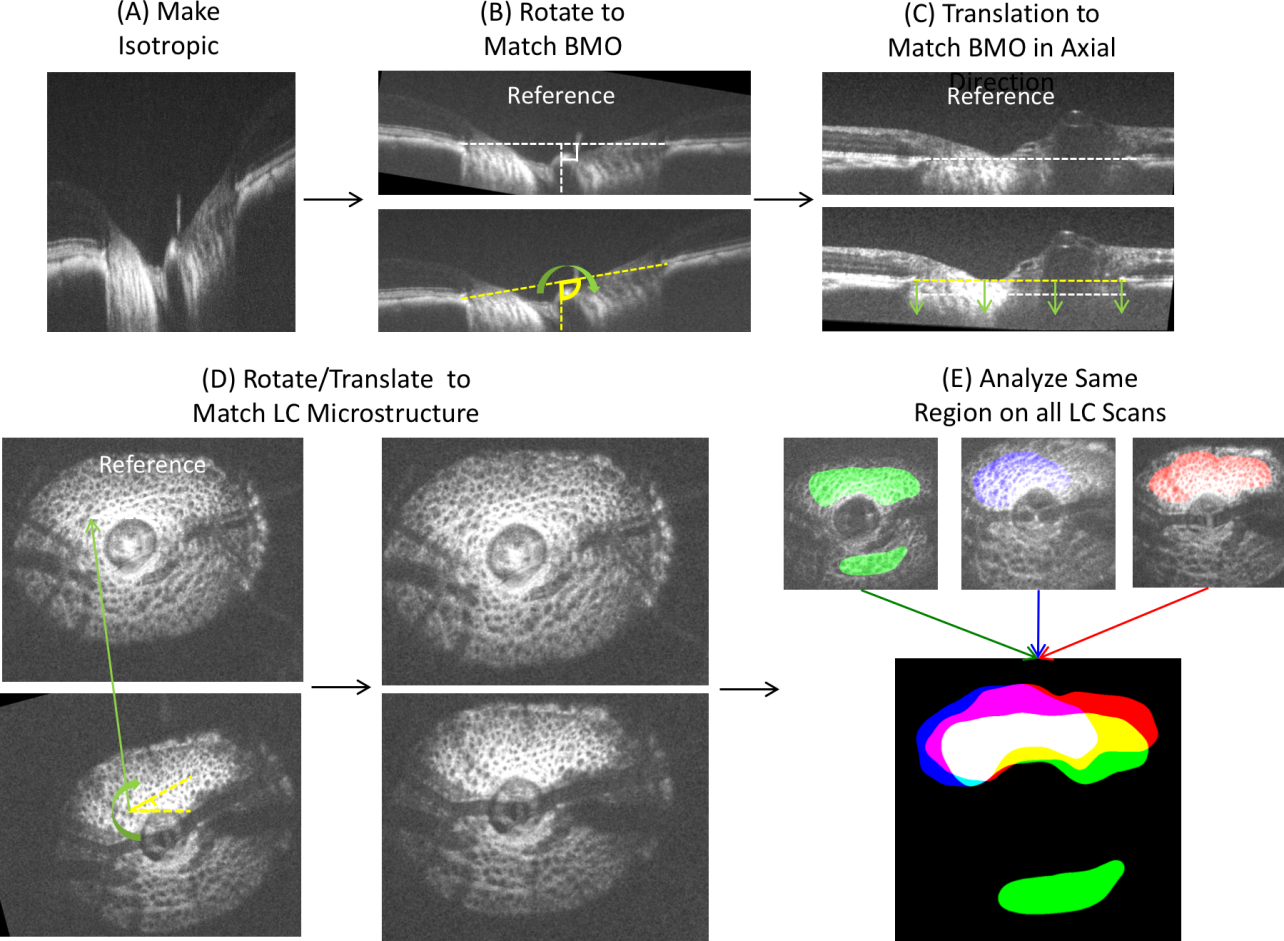
Image analysis procedure. (A) Images were adjusted for isotropic dimensions, (B) and rotated to match the angle of Bruch membrane opening (BMO). (C) Images were translated in the axial direction to match the axial height of the BMO. (D) The microstructures were aligned manually via 3D rotation and translation. (E) Visible LC was denoted and a common overlapping region (white color region) was used for analysis.

Qualified images were segmented using a previously described 3D automated segmentation algorithm of LC microstructure to quantify beam thickness, pore diameter and beam thickness to pore diameter ratio.[17,20] While we corrected for optical magnification by comparing with histology, we included beam thickness to pore diameter ratio in our analysis because it offered the ability to eliminate any magnification confounders (the ratio remains the same despite small changes in magnification between eyes). The thickness at a given point in the beam is defined as the radius of the largest sphere containing that point within the segmented volume. All the thickness values are averaged to provide a global mean. This method was previously described and implemented in ImageJ.[21] Prior to segmentation, bicubic interpolation (ImageJ) was used to make the image isotropic. Because the visible LC varied between images acquired in the various pressure settings, the analysis was performed only in overlapping regions visible in all images to prevent the confounding effect related to quantification of different areas of the LC. Images were registered by rigid-body translation and rotation in 3D to align the LC microstructure, as outlined in Fig 2. The BMO was lined up in two orthogonal slices and verified on multiple other slices. Alignment was performed by matching LC microstructure such as pores.

### Histological processing for OCT scaling and verification

Since eyes vary in optical power and OCT systems are optimized for imaging human eyes, OCT images of primate ONHs have to be rescaled in the transverse dimensions.[9,22] Histology was used to obtain the eye-specific transverse scales for OCT images, which was done for all primates. Histology was also used to verify the depth penetration and microstructure of the LC beams and pores observed in OCT images, which was done for Monkey 2.

Before sacrificing one of the animals (Monkey 2), the anterior chambers were cannulated with a 27-gauge needle and IOP was set at 30 mmHg (OD) and at 5 mmHg (OS) via gravity-based perfusion. Within 30 minutes of death, both eyes were enucleated and processed for histology. The eyes were then immersed in 10% formalin for 24 hours while IOP was maintained. The posterior poles containing the ONH region were excised using an 8 mm trephine, immersed in a cryoprotectant, 30% sucrose, for 24 hours, then cryosectioned at 30μm thickness. Both eyes were cryosectioned and analyzed with histology. The OCT-scanned eye (OD) was cryosectioned coronally to confirm the in-vivo microstructures of the LC visualized in OCT with histology. The contralateral non-OCT-scanned eye was cryosectioned axially to confirm the in-vivo penetration of OCT signal throughout LC thickness, assuming small difference between contralateral eyes.[23]

Histological sections were imaged with polarized light microscopy [24] on a stereo dissecting microscope (SMZ1500, Nikon Instruments Inc., Melville, NY, 16-bit greyscale, 0.765 μm/pixel). Ex-vivo images were then manually registered into 3D stacks based on ONH anatomical landmarks, including scleral canal shape, central retinal blood vessels and vessel trunk, as well as major LC collagen beams that can be detected repeatedly in adjacent sections.[25] The 3D OCT volumes were rotated and resliced in ImageJ [26] to find matching LC microstructures of beams and pores between in-vivo OCT and histological sections. LC thickness was computed by multiplying the number of OCT C-mode or coronal histology sections that had LC with the thickness of each OCT or histology section. A confidence interval for the measurements of thickness was derived from the distance between adjacent coronal images, which for the OCT volumes was the axial resolution, and for the histology was the thickness of the histological sections.

The histological processing and analysis described above were also performed on the OCT-scanned eye in each monkey and the coronal scleral canal dimensions were manually obtained from the 3D stacks and used to set the transverse scale of the OCT volumes. Histology was used to guide assessment of the posterior LC, which was defined as a drop-off in reflectivity of the LC on OCT in the transition area.

Note that no stains or labels were used and the tissues were never dehydrated with ethanol or embedded in paraffin or plastic. In a previously published study using 6 porcine eyes, we found that the fixation, sectioning, imaging and 3D reconstruction caused only minimal tissue shrinkage or deformation, with average changes compared with the fresh tissues smaller than 3%.[27]

### Reproducibility

The reproducibility of the segmentation analysis and quality grading was assessed by measuring the imprecision standard deviation of repeated measurements using a measurement error model. The relative imprecision was computed by dividing imprecision standard deviation by the average measurement. Low imprecision indicated high reproducibility.

### Statistical analysis

Random intercept linear mixed effects models were used to determine the effect of the various pressures and image quality on LC microstructure.[28] The models accounted for repeated measurements in each eye as well as the autocorrelation between eyes of the same animal. IOP, ICP, translaminar pressure difference (TLPD; IOP-ICP), and image quality were modeled as a predictor of LC microstructure. Various combination of the variables and their interaction were tested (Table 1). An interaction term between TLPD and ICP allow to account to the actual pressure level and not only to the pressure difference as the same pressure difference might occur in various pressure levels.

**Table 1:** Comparison of the various models that were tested. Lower Akaike information criterion (AIC) denotes the better model. * denotes interaction term between the variables. Bold denotes best performing model as judged by AIC.

Model	AIC
TLPD + quality	-293.7
IOP + quality	-293.1
ICP + quality	-293.2
IOP * ICP + quality	-290.1
IOP * ICP * quality	-286.8
TLPD * IOP + quality	-289.7
TLPD * ICP + quality	**-300.8**
TLPD * IOP * quality	-284.6
TLPD * ICP * quality	-297.4

Akaike information criterion (AIC), a numerical method of balancing between model fit and model complexity, was used to select the better model where the model with lower AIC being superior. A p < 0.05 was considered as statistically significant. R Language and Environment for Statistical Computing program (version 3.1.1), was used for the statistical analysis.[28]

## Results

### Subjective detection of LC microstructure changes caused by pressures modulation

**Fig 3:**
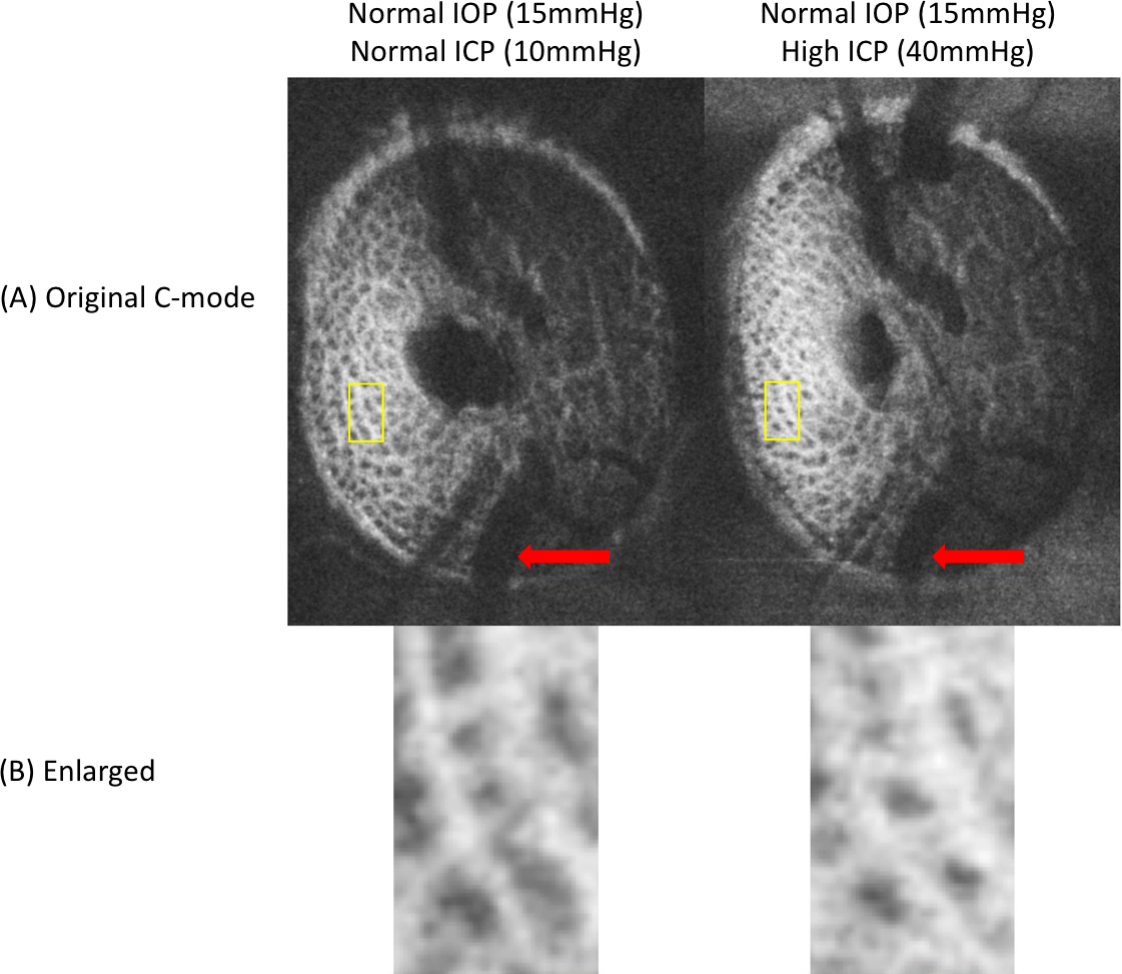
Example of variations in lamina cribrosa (LC) microstructure with pressure modulation. (A) enface images show variation in the vasculature (red arrows) with differences in intracranial pressure. (B) Matching regions of the LC also feature prominent changes in LC microstructure, with decreased pore diameter and beam thickening observed with higher intracranial pressure (ICP; right), at a fixed intraocular pressure (IOP).

Subjective assessment of the LC microstructure demonstrated change with respect to both IOP and ICP. An example of the variation in LC microstructure parameters with changes in ICP is shown in Fig 3. In this eye, increasing ICP led to an increase in beam thickness and decrease in pore diameter.

### Correspondence between OCT and histology in assessment of LC microstructure

**Fig 4:**
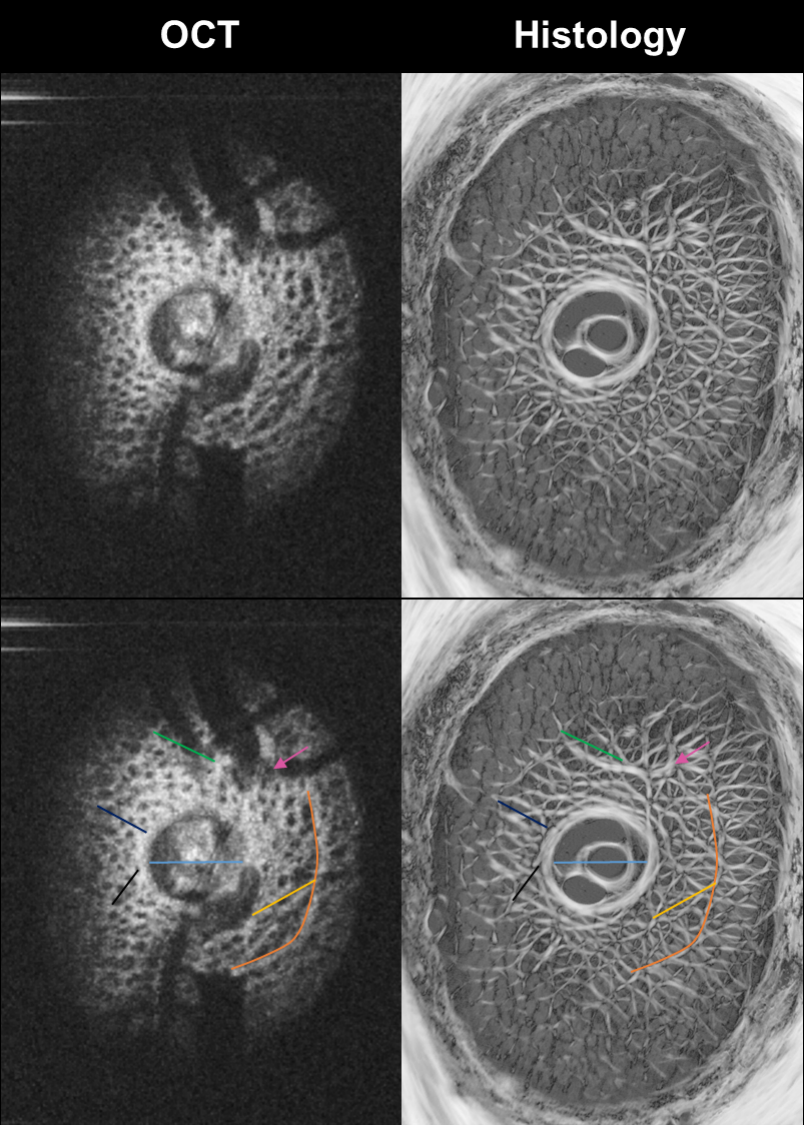
Matching LC microstructure between enface OCT images (left column) and histology (right column). Color lines and arrow (bottom row) were added to illustrate some of the corresponding ONH structures. Note the similarity in structures discernible with both techniques, including the details of collagen beams and pores of the LC.

The LC is a complex 3D microstructure, which can be imaged in-vivo using OCT.[29] We validated the ability of OCT to capture the LC total thickness and the microstructure appearance by comparing our OCT findings to histology of both eyes of one animal (M2). Comparable LC thickness were measured, with an average of 335 ± 3.9 μm of in-vivo OCT image and 330 ± 30 μm of histological image. Larger variability in histology measurements, as reflected by higher standard deviation, reflects the larger axial distance between consecutive histological sections compared to OCT axial resolution. Similarity in LC microstructures, including collagen beams and pores, were detected throughout the LC up to the very posterior LC (Fig 4). Histology was used to calibrate OCT lateral and axial dimensions.

### Repeatability

The repeatability of the subjective assessment of image quality had a good relative imprecision of 9.01%. It is important to note that when the two graders did differ in their subjective grading of the images, the difference between grading never exceeded a value of one. 20 randomly selected scans were regraded by one of the graders (BW). Intraobserver relative imprecision was 5.96%. The repeatability of the automated segmentation measurements was excellent, with all LC microstructure parameters having a relative imprecision SD less than 5%: beam thickness = 2.03%, pore diameter = 1.76%, and beam-pore ratio = 4.55%.

### Quantitative LC microstructure changes caused by pressures modulation

When the effects of IOP on LC microstructure were assessed, without considering ICP, there was no statistically significant correlation between the two (beam thickness vs IOP–p = 0.24, pore diameter vs IOP–p = 0.27, beam thickness to pore ratio vs IOP–p = 0.74). Furthermore, when assessing the effect of ICP, without considering IOP, we found no significant correlation with LC microstructure.

**Fig 5:**
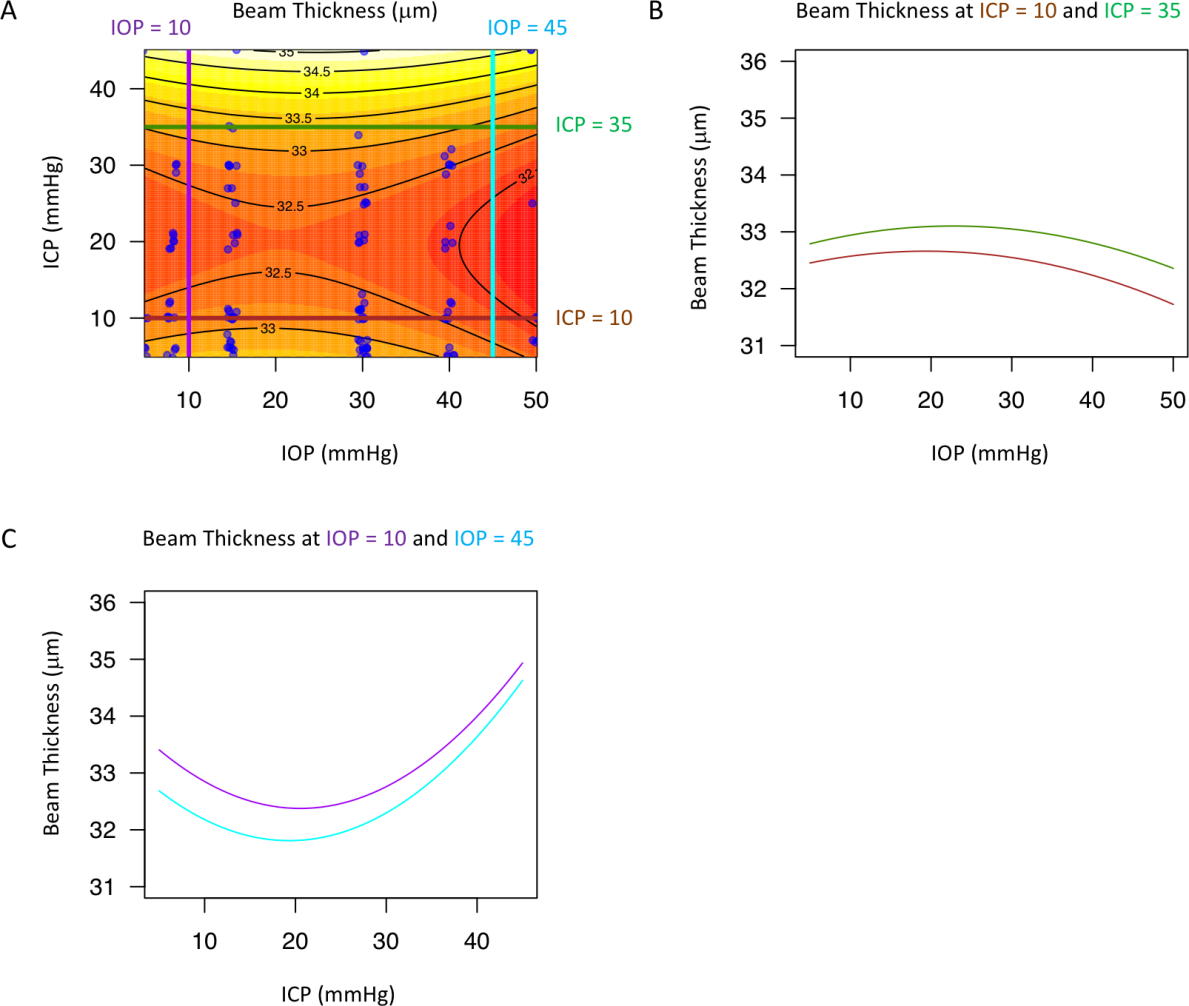
Change in lamina cribrosa (LC) beam thickness with intraocular (IOP) and intracranial pressure (ICP). (A) Contour plot showing change in beam thickness as a function of IOP and ICP. Black lines indicate the contour line at the same beam thickness. Blue dots indicate actual measurements acquired in the experiments. A sample of the contour plot at a set of (B) fixed ICP (ICP = 10mmHg, brown line; ICP = 35mmHg, dark green) and (C) fixed IOP (IOP = 10mmHg, purple; IOP = 45mmHg, light blue) conditions demonstrate the complex interaction between IOP and ICP on beam thickness.

**Fig 6:**
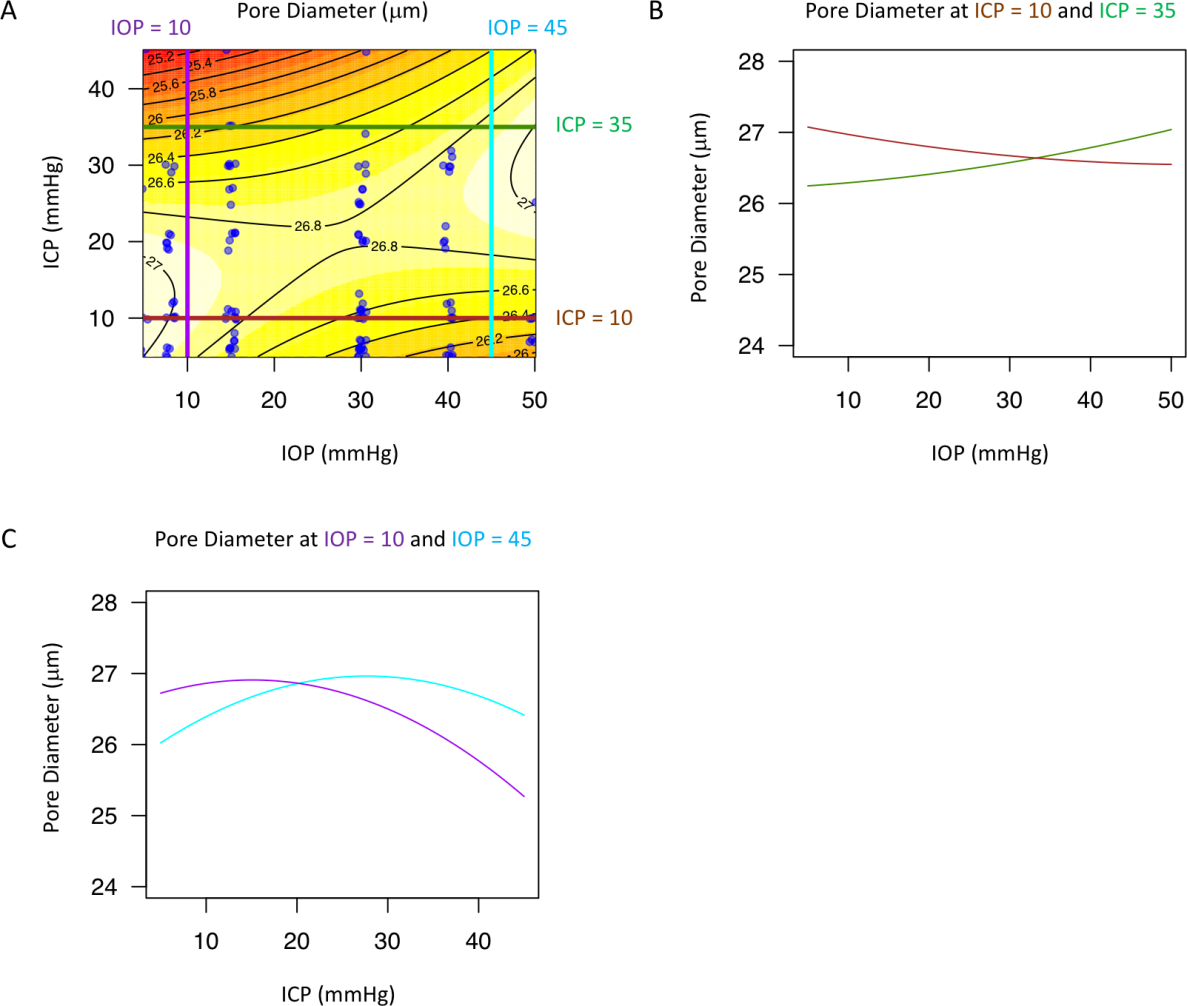
Change in lamina cribrosa (LC) pore diameter with intraocular (IOP) and intracranial (ICP) pressure. (A) Contour plot showing change in beam pore ratio as a function of IOP and ICP. Black lines indicate the contour line at the same pore diameter. Blue dots indicate actual measurements acquired in the experiments. A sample of the contour plot at a set of (B) fixed ICP (ICP = 10mmHg, brown line; ICP = 35mmHg, dark green) and (C) fixed IOP (IOP = 10mmHg, purple; IOP = 45mmHg, light blue) conditions demonstrate the complex interaction between IOP and ICP on pore diameter.

**Fig 7:**
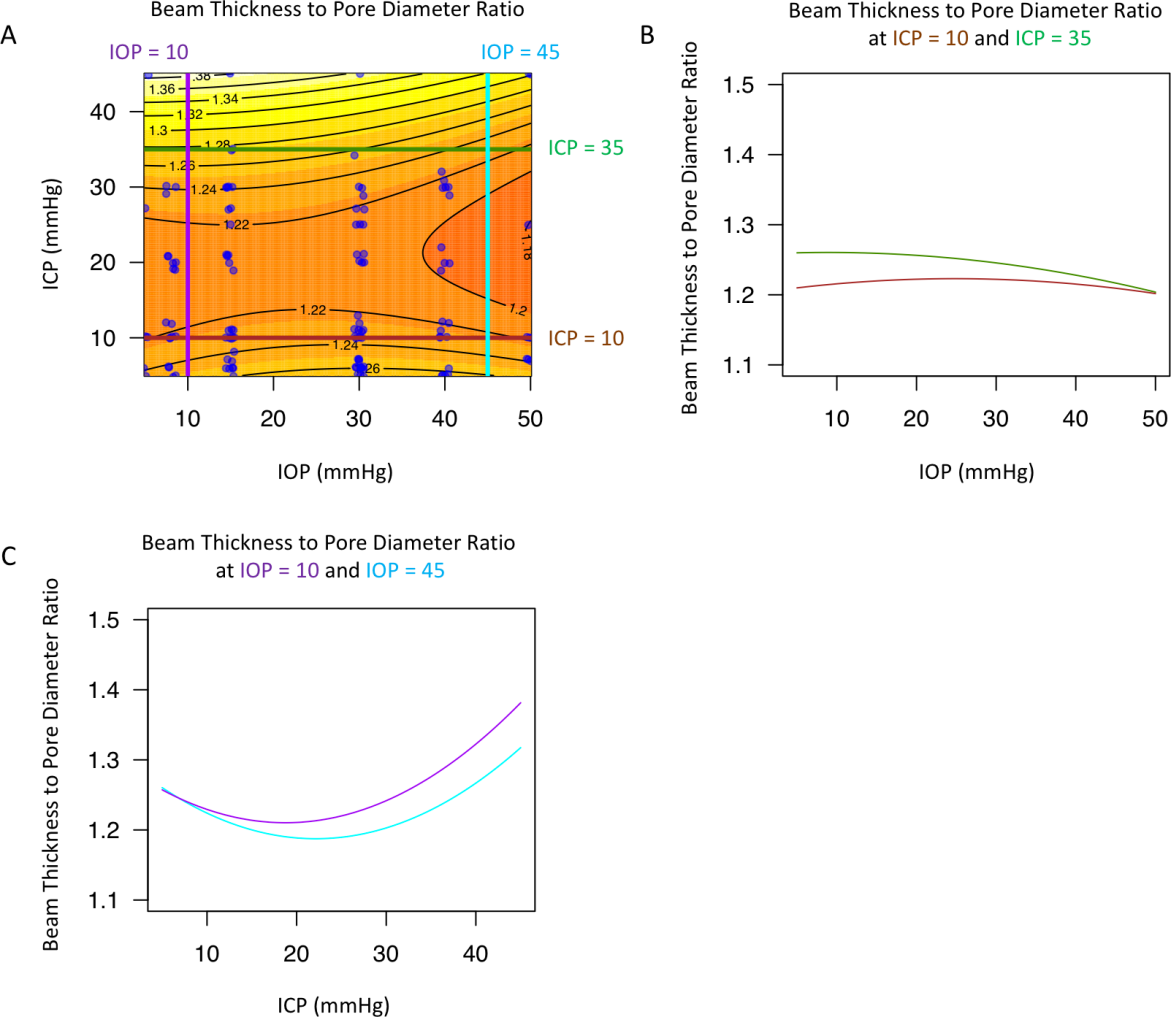
Change in lamina cribrosa (LC) beam thickness to pore diameter ratio with intraocular (IOP) and intracranial (ICP) pressure. (A) Contour plot showing change in beam pore ratio as a function of IOP and ICP. Black lines indicate the contour line at the same beam thickness to pore diameter ratio. Blue dots indicate actual measurements acquired in the experiments. A sample of the contour plot at a set of (B) fixed ICP (ICP = 10mmHg, brown line; ICP = 35mmHg, dark green) and (C) fixed IOP (IOP = 10mmHg, purple; IOP = 45mmHg, light blue) conditions demonstrate the complex interaction between IOP and ICP on beam pore ratio.

The best models describing the effect of IOP and ICP on the LC microstructure featured both ICP and TLPD, with an interaction between the two (Table 1). The effect of IOP and ICP on beam thickness (Fig 5), pore diameter (Fig 6), and beam thickness to pore diameter ratio (Fig 7) was non-linear and changed with level of the measured parameter. The models for all three parameters included statistically significant interaction between IOP and ICP, which can be appreciated when the effect at a given pressure is plotted against the varying effect of the other pressure showing a non-linear contour (Fig 5B and 5C; Fig 6B and 6C; Fig 7B, and 7C). Furthermore, the lines were not parallel highlighting the varying effect of pressure level.

## Discussion

This study represents the first in-vivo characterization of the acute effects of IOP and ICP on the 3D LC microstructure. We demonstrate the ability to visualize in-vivo and analyze LC microstructure changes occurring because of altered IOP and ICP. While previous studies provide epidemiologic evidence of a potential link in the role of IOP and ICP with disease,[6,11–13] our study demonstrate in an in-vivo model that the LC microstructure acutely deforms in accordance to a complex interaction between IOP and ICP.

**Fig 8:**
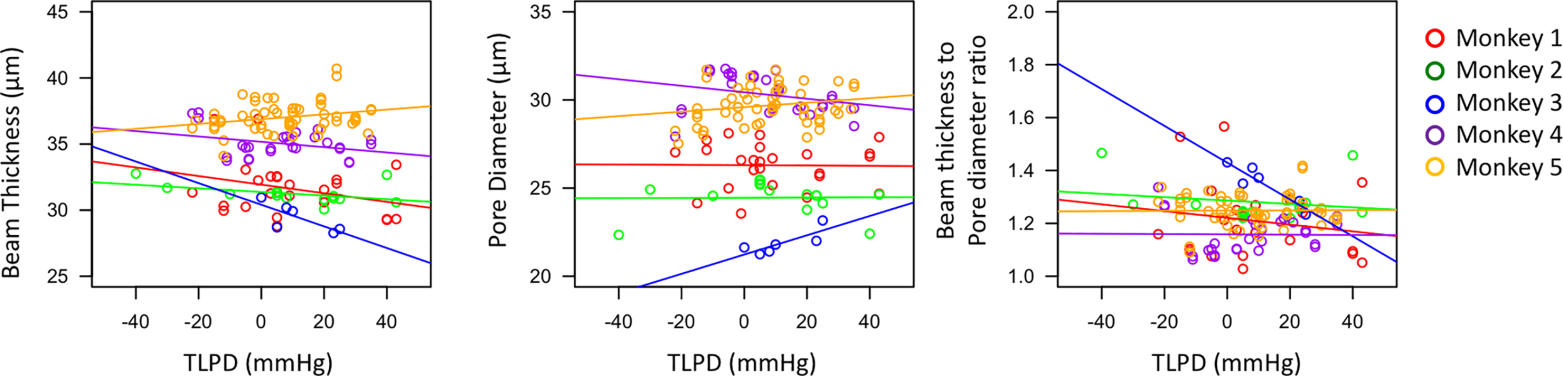
Monkey specific response to pressure modulation. Scatterplot of beam thickness, pore diameter, and beam thickness to pore diameter ratio versus translaminar pressure difference (TLPD) for the 5 monkeys (color-coded). Each line indicated the line of best fit to help illustrate the trend.

The microstructural changes in LC with IOP we report are in the same magnitude (~5–10%) as previously modeled[30] and in ex-vivo models.[16] Both previous studies noted heterogeneity in regional LC respond to stress, hence the importance of evaluating the same region across all pressure conditions. As expected, a marked difference in the response to pressure modulations was noted between animals (Fig 8), which may reflect the individual biomechanical properties of each subject’s LC. This highlights the importance of analyzing eye-specific response to changes in pressure, rather than pooling across animals. Furthermore, it is required to develop methods to determine biomechanical properties of individual eyes to assess their response to pressure insults.[31] The variability of the LC microstructure’s response to changes in IOP and ICP may help explain the variability in response to these pressures in diseases such as glaucoma[32] and intracranial hypertension.[33]

A primary finding of this paper is that it is crucial to consider both IOP and ICP when assessing LC microstructure. Our models that included both IOP and ICP were superior, based on AIC analysis (Table 1), in assessing LC microstructure deformation. This finding is especially significant because AIC inherently tends to prefer simpler models (in our case, those including only IOP or ICP). The best performing model (TLPD interacting with ICP) has all the characteristics we would hypothesize to be important: an interaction between TLPD and ICP, as well as the presence of both IOP and ICP information in the form of TLPD. This finding is in agreement with a previous study that demonstrated that TLPD is a better predictor of optic nerve head surface changes than either IOP or ICP.[15] Our results emphasize that the conventional standard of assessing the effect of IOP only in glaucomatous eyes may not be adequate. Given the strong interaction between the two pressures, we can only assess the way IOP affects the LC while considering the ICP. Further investigation is required to elucidate how these LC microstructural changes affect vision.

Interestingly, there is a region around 10-30mmHg ICP where there is relatively little change in LC microstructure with IOP (Figs 5, 6 and 7). This ICP range is normal or slightly above normal in cerebrospinal fluid opening pressure in primates.[34] This pattern of minimal change in LC microstructure was not consistently observed with IOP for changes in ICP. The relative stable reaction to increased translaminar pressure difference at a level around normal ICP could indicate that the eye is better suited for alterations in IOP rather than fluctuations in ICP. This may be due to the exposure of the eye to frequent occasions of elevated intraocular pressure, such as from blinking, rubbing of the eyeball, and other natural causes of elevated IOP. However, as ICP rises, increases in IOP result in much greater deformation at the level of the LC microstructure.

Increasing ICP beyond the normal pressure range (while keeping IOP within normal ranges) is generally associated with beam thickening and shrinking of the pores (Figs 5 and 6), which may be explained by ICP acting concentrically in the sub-arachnoid space around the optic nerve. These microstructural changes can lead to strangulation of the axoplasmic flow, consistent with previous models demonstrating impaired axoplasmic transport at the level of the LC in animal models of papilledema.[5] These changes may contribute to the swelling and substantial deformation of the ONH tissues associated with intracranial hypertension.[35]

The effect of increasing IOP exhibited much more complexity. Increasing IOP leads to a general decrease in beam thickness, which is expected as increasing tension on the eye would create tension across the LC, making beams thinner. However, the effect of increasing IOP on pore diameter leads to two different effects (Fig 6). At normal ICP (10 mmHg, brown lines), increasing IOP leads to a decrease in pore diameter. However, at high ICP, increasing IOP leads to an increase in pore diameter. This finding is the outcome of the interaction between the pressures as the effect of IOP on the eye depends on the actual ICP setting. It is possible that at high ICP there is high circumferential pressure to constrict the LC. Therefore, the LC can only stretch in response to increasing IOP, causing the pores to increase in size. However, at normal ICP, without this excessive external circumferential pressure, increasing IOP causes compression of the LC pores (Fig 6).

In our previous study of LC microstructure in healthy and glaucomatous human eyes,[17] we reported that glaucomatous eyes had thicker beams and smaller pores compared to healthy eyes. These seemingly conflicting findings between the glaucomatous eyes and our current findings (beam thinning with increase IOP at normal ICP) using the acute IOP modulation likely reflect remodeling of the LC over time or even LC collapse under chronic pathologic levels of strain. We hypothesize that chronic exposure to elevated IOP with normal ICP leads to collagen fiber recruitment to negate the pressure effect with thicker beams, as suggested in a previous study.[36] However, in acute experiments, changes in the LC microstructure likely result from expansion of the globe when IOP is elevated in comparison with ICP, causing beams to stretch. There are various mechanisms through which beam thinning could lead to deleterious effects. First, the beam stretching could result in activation of the astrocytes along with biological responses such as extracellular matrix remodeling.[37,38] Second, damage from acute deformation may also have a vascular component. Capillaries pass through the LC beams, nourishing the axonal bundles passing through.[39] Beam strain and thinning could compromise perfusion and nourishment to the axon bundles, especially given the expansion of axonal pores.

Our study has several limitations that should be considered. The acute experimentation does not provide information on the effect of remodeling on the microstructure of the LC. However, our data provide the basis to determine, in future projects, the contribution of tissue remodeling to the response to pressure modulations. Understanding the mechanisms leading to chronic visual loss requires a systematic characterization of the acute response of the LC to IOP and ICP, as it is the consequence of these acute changes that result in chronic responses such as astrocyte activation and extracellular matrix remodeling. Another limitation to consider is that our analysis is based only on the visible lamina as captured with OCT. Considering the large vasculature in the superficial ONH, there are large areas in the LC that are shadowed by the vessels. This inevitable limitation of the data obtained with OCT technology is mostly mitigated when pooling data across animals as the visible lamina varies. It should be emphasized that our study focused only on the structural outcome of pressure modulation without considering the functional consequences, which is a matter of future studies. Our experimental design included 5 minutes pause following each pressure modulation. This time interval was chosen to balance between dissipation of the viscoelastic effect of the tissue and the overall duration of the experiment with the ensuing deterioration in image quality with time. Further studies are required to assess the viscoelastic effects of IOP and ICP on the LC to identify the optimal time between pressure condition changes. Another potential limitation to consider is that our statistical approach was geared toward identifying differences in LC microstructure with changes in IOP and ICP, not to predict the mechanical deformations. A linear model can be proven useful to capture associations between parameters, even when mechanical deformations are well known to be nonlinear. We opted to use a simpler and easier to interpret model instead of a complex non-linear model. Future work can take advantage of more complex non-linear models. In this study, we were also limited by the sample size; larger numbers of primates would have allowed for assessment of more complex non-linear models. However, we do note that we were able to achieve statistical significance despite limited number of primates. Finally, tissue processing may create artifacts such as shrinking which may influence our ability to calibrate our ability to calibrate OCT measurements. However, elsewhere we have shown[27] that for the tissue processing methods used in this study the effects are very small (on average under 3%), and therefore these are unlikely to affect the conclusions in this work.

In summary, we demonstrate in-vivo that LC microstructure deforms as a response to acute alterations in both IOP and ICP. Due to the interaction between IOP and ICP, it is important to consider both IOP and ICP when accurate investigation of the LC response to either pressure is sought.

## Supporting information

S1 Table Raw data by primate. Raw data containing information regarding the monkey, intraocular pressure (IOP), intracranial pressure (ICP), beam thickness to pore diameter ratio (beam pore), beam thickness (beam), pore diameter (pore) and eye (OD–right, OS–left).
(CSV)

S1 Fig **Example of LC images of (A) quality = 1, (B) quality = 2, and (C) quality = 3.** The worst quality scans (quality = 1) had visible LC beams and pores, but without a clear transition from beams to pores. The best quality scans (quality = 3) had very well defined pore structures as well as a easily delineated transition point from beams to pores.
(TIF)

S2 Fig Image quality metrics. Histogram of (A) IOP setting per image quality (1 –worst quality, 3 –best quality) and (b) ICP setting per image quality (C) image quality per monkey.
(TIF)
