# Supplementary figures and images for: In-vivo effects of intraocular and intracranial pressures on the lamina cribrosa microstructure
Source: PLoS One. 2017 Nov 21;12(11):e0188302. doi: 10.1371/journal.pone.0188302 (PMC5697865; doi:10.1371/journal.pone.0188302)

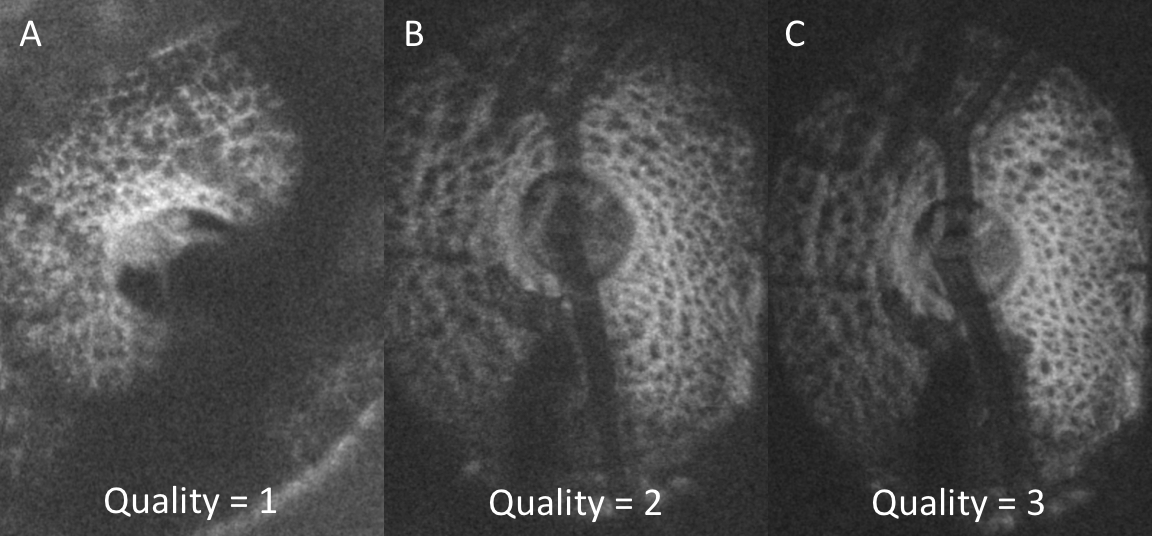

Supplement: S1 Fig — Example of LC images of (A) quality = 1, (B) quality = 2, and (C) quality = 3. The worst quality scans (quality = 1) had visible LC beams and pores, but without a clear transition from beams to pores. The best quality scans (quality = 3) had very well defined pore structures as well as a easily delineated transition point from beams to pores. (TIF) [file pone.0188302.s002.tif]

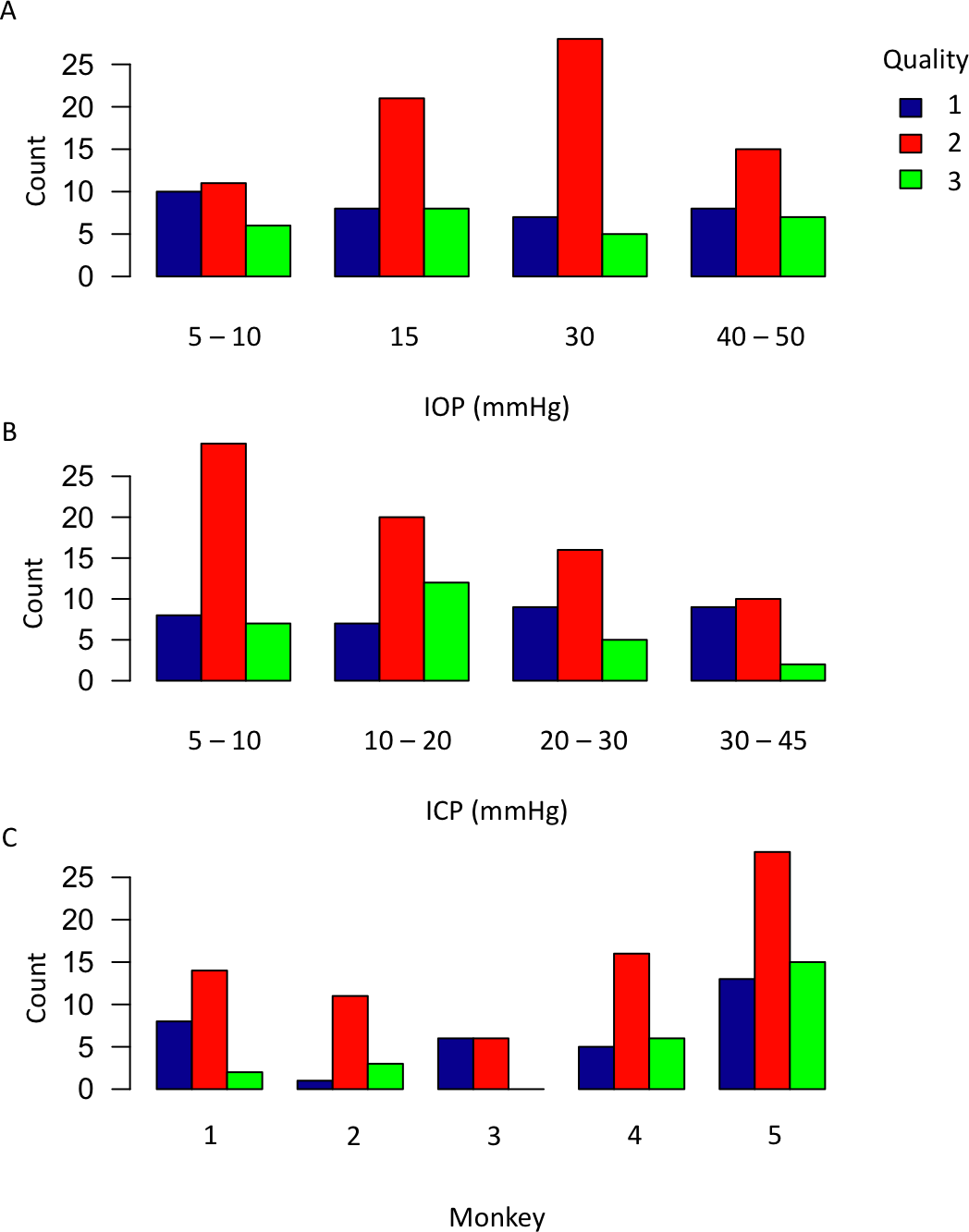

Supplement: S2 Fig — Histogram of (A) IOP setting per image quality (1 –worst quality, 3 –best quality) and (b) ICP setting per image quality (C) image quality per monkey. (TIF) [file pone.0188302.s003.tif]
